# Applying artificial neural network in predicting sepsis mortality in the emergency department based on clinical features and complete blood count parameters

**DOI:** 10.1038/s41598-023-48797-9

**Published:** 2023-12-05

**Authors:** Beata Pui Kwan Wong, Rex Pui Kin Lam, Carrie Yuen Ting Ip, Ho Ching Chan, Lingyun Zhao, Michael Chun Kai Lau, Tat Chi Tsang, Matthew Sik Hon Tsui, Timothy Hudson Rainer

**Affiliations:** 1 Department of Emergency Medicine, School of Clinical Medicine, Li Ka Shing Faculty of Medicine, The University of Hong Kong, Hong Kong Special Administrative Region, China; 2 Accident and Emergency Department, Queen Mary Hospital, Hospital Authority, Hong Kong Special Administrative Region, China

**Keywords:** Infectious diseases, Prognosis

## Abstract

A complete blood count (CBC) is routinely ordered for emergency department (ED) patients with infections. Certain parameters, such as the neutrophil-to-lymphocyte ratio (NLR), might have prognostic value. We aimed to evaluate the prognostic value of the presenting CBC parameters combined with clinical variables in predicting 30-day mortality in adult ED patients with infections using an artificial neural network (ANN). We conducted a retrospective study of ED patients with infections between 17 December 2021 and 16 February 2022. Clinical variables and CBC parameters were collected from patient records, with NLR, monocyte-to-lymphocyte ratio (MLR), and platelet-to-lymphocyte ratio (PLR) calculated. We determined the discriminatory performance using the area under the receiver operating characteristic curve (AUROC) and performed a 70/30 random data split and supervised ANN machine learning. We analyzed 558 patients, of whom 144 (25.8%) had sepsis and 60 (10.8%) died at 30 days. The AUROCs of NLR, MLR, PLR, and their sum were 0.644 (95% CI 0.573–0.716), 0.555 (95% CI 0.482–0.628), 0.606 (95% CI 0.529–0.682), and 0.610 (95% CI 0.534–0.686), respectively. The ANN model based on twelve variables including clinical variables, hemoglobin, red cell distribution width, NLR, and PLR achieved an AUROC of 0.811 in the testing dataset.

## Introduction

Sepsis is a global health burden. In 2017, an estimated 48.9 million cases and 11 million related deaths occurred worldwide, with 85% of the cases in low- or middle-income countries^1^. Identifying sepsis and patients who are at risk of deterioration early in the emergency department (ED) is essential for prompt intervention to prevent morbidity and mortality. The quick Sequential Organ Failure Assessment (qSOFA) score is used in many non-intensive care settings to identify high-risk patients with infections. A meta-analysis showed that it has a high pooled specificity of 83%, but its pooled sensitivity was only 51% in predicting in-hospital mortality^2^. The Third International Consensus Definitions for Sepsis and Septic Shock (Sepsis-3) recommend to identify organ dysfunction in septic patients by an acute change in the Sequential Organ Failure Assessment (SOFA) score of ≥ 2^3^. However, based on the worst values within 48 h of admission and a cutoff score of > 9, the sensitivity and specificity for in-hospital mortality were only 65.8% and 75.5%, respectively^4^. A more reliable, fast, and affordable method is needed to identify at-risk septic patients in the ED.

The complete blood count (CBC) contains important information on the hematological system whose value in sepsis prognostication might have been overlooked^5^. The CBC has multiple potential advantages in identifying sepsis and predicting mortality: a low cost that enables its wider adoption in resource-poor settings, a shorter turnaround time than liver and renal function tests to support early clinical decisions, and good availability since it is often done as a routine workup for hospitalized patients. In daily practice, clinicians use elevated white blood cell (WBC) counts to gauge the severity of infection and platelet count to calculate the SOFA score. However, the CBC has not been routinely used in the ED as an objective tool in sepsis prognostication.

Previous studies have demonstrated the prognostic value of several CBC parameters in sepsis. The initial hemoglobin level on ED admission is associated with 90-day mortality^6^, and hematocrit in the first 24 h in the intensive care unit (ICU) is associated with increased 30-day mortality^7^. Increased baseline red cell distribution width (RDW), an erythrocyte index reflecting the heterogeneity in the size of circulating erythrocytes, is associated with sepsis mortality^8^. The lowest platelet count in the first 72 h after ICU admission is significantly lower in septic non-survivors^9^. Meta-analyses have shown that the neutrophil-to-lymphocyte ratio (NLR) and platelet-to-lymphocyte ratio (PLR) are significantly higher in septic non-survivors^10,11^. In surgical ICU settings, combining mean platelet volume to platelet count ratio (MPV/PC), NLR, monocyte-to-lymphocyte ratio (MLR), and PLR significantly improved mortality prediction^12^. A scoring system based on RDW, delta neutrophil index (DNI), and MPV/PC has been shown to be more useful than platelet count alone in predicting sepsis mortality^13^.

Literature combining patient characteristics, clinical variables, and CBC parameters in sepsis prognostication is scarce. The complex relations between these factors may require non-linear data modeling in prognostication and machine learning methods, such as artificial neural networks (ANNs). However, the use of ANNs in sepsis prognostication based on these parameters has not been evaluated. The objectives of this study were to (1) compare the patient characteristics, triage vital signs, and CBC parameters of adult ED patients with infections who did and did not survive up to 30 days and (2) evaluate the prognostic value of CBC parameters combined with patient characteristics and triage vital signs in predicting 30-day all-cause mortality for such patients using an ANN.

## Methods

### Study design

This was a retrospective observational study of consecutive adult patients presenting to the ED of Queen Mary Hospital (QMH) with an infection over 3 months from 17 December 2021 to 16 February 2022. QMH is a university-affiliated tertiary hospital in Hong Kong with 1,700 beds and an annual ED attendance of 100,000.

The study was approved by the Institutional Review Board of The University of Hong Kong/Hospital Authority Hong Kong West Cluster (HKU/HA HKW IRB; reference number: HKWC-2023-452) and conducted in full compliance with the Declaration of Helsinki and local research ethics guidelines. Informed consent was waived by the HKU/HKW IRB because this was a retrospective study and patients remained anonymous during the data analysis. We followed the Strengthening the Reporting of Observational studies in Epidemiology (STROBE) guidelines^14^ and Transparent Reporting of a multivariable prediction model for Individual Prognosis or Diagnosis (TRIPOD) guidelines^15^ in reporting the findings of this study.

### Study population

All patients who were aged ≥ 18 years and presented to the QMH ED with a clinical diagnosis of infection during the study period were recruited. All clinical diagnoses and decisions on laboratory tests, including CBCs, were made by the treating clinical teams. For patients with more than one ED attendance for infections within the study period, only the first ED presentation was included as the index presentation to avoid duplication.

The exclusion criteria were (1) patients who did not have a CBC collected within 48 h of ED presentation; (2) patients younger than 18 years old because of different normal ranges of CBC parameters in children and adults^16^; (3) patients who were subsequently diagnosed with conditions other than an infection after hospital workup; (4) patients with pre-existing hematological malignancies or myeloproliferative disorders because these conditions affect CBC cell counts^17^; (5) patients who had received chemotherapy in the past 12 months, which might also affect the cell counts; and (6) patients who had received blood product transfusions in the previous 2 weeks.

### Data collection

We collected the following data: (1) patient characteristics, including age, sex, old age home resident status, and Charlson Comorbidity Index (CCI); (2) ED triage vital signs, including temperature, systolic blood pressure (SBP), diastolic blood pressure (DBP), mean arterial pressure (MAP), pulse rate, respiratory rate (RR), and oxygen saturation (SpO_2_); (3) pathogens and organ systems involved in infection, by reviewing the results of microbiology tests on patients’ blood, sputum, urine, and other specimens; (4) complications, including organ dysfunction; and (5) clinical outcomes, including ICU admission and 2-, 7-, 30-, and 90-day mortality.

We extracted nine CBC parameters at presentation, comprising the total WBC, neutrophil, lymphocyte, monocyte, basophil, and platelet counts, hemoglobin, hematocrit, and RDW, and calculated the NLR, MLR, and PLR accordingly. We calculated the SOFA score based on the worst clinical and laboratory parameters within 48 h of ED presentation^18^. For patients with missing partial pressure of arterial oxygen (PaO_2_) data, we determined the respiratory component of the SOFA score based on the method described by Grissom et al.^19^ that replaces PaO_2_ with SpO_2_.

The data were collected by reviewing the electronic medical records in the Clinical Management System (CMS) of the Hospital Authority (HA). The HA CMS is a central repository of all clinical records of patients attending public EDs and hospitals. It is also connected to the central death records in Hong Kong; therefore, the chance of missing mortality was very low. We reviewed the whole clinical course of each case from ED presentation to hospital discharge or death. All data was collected by trained research personnel using a standardized data collection spreadsheet and cross-checked by the first two authors.

### Patient outcome

The primary outcome was 30-day all-cause mortality.

### Statistical analysis

A sample size was not calculated because of the lack of similar studies to guide such a calculation. Missing data on vital signs were imputed using the vital signs documented on hospital admission, if available. We did not impute missing laboratory or outcome data.

The distribution of characteristics of the study population was analyzed using descriptive statistics, with categorical variables reported as numbers and proportions and continuous variables as mean +/− standard deviation (SD) or medians with interquartile ranges (IQR), as appropriate. We then conducted univariate analysis, with the distribution of categorical variables compared between 30-day survivors and non-survivors using Pearson’s chi-squared (χ^2^) test or Fisher’s exact test and continuous variables with Student’s t-test or the Mann–Whitney U test, as appropriate. The discriminatory performance of the NLR, MLR, and PLR in predicting the primary outcome was evaluated using the area under the receiver operating characteristic curve (AUROC). Using the approach of Djordjevic et al., we further evaluated the prognostic performance of the sum of NLR, MLR, and PLR to determine whether the sum would surpass the individual ratios alone^12^.

We then performed supervised machine learning using an ANN model with a multi-layer perceptron. The dataset was randomly split in a 70/30 ratio into a training set and a testing set. The following 12 variables were input into the model to predict the primary outcome: patient age, sex, old age home resident status, CCI, triage MAP, pulse rate, RR, SpO_2_, hemoglobin level, RDW, NLR, and PLR from the first CBC taken after the ED presentation, since they were significantly associated with 30-day mortality in univariate analyses. We did not input hematocrit, total WBC, neutrophil, and lymphocyte counts into the model to avoid redundancy, although they were also significantly associated with 30-day mortality in univariate analyses. All continuous variables were normalized before being entered into the model. The ANN was built with one hidden layer and seven hidden nodes and was trained using backpropagation with gradient descent. The trained ANN model was then applied to the testing dataset to evaluate its discriminatory performance. We determined the accuracy and the AUROC of the derived ANN model in the testing set.

All data were analyzed with SPSS for Windows version 27.0 (IBM Corp., Armonk, NY, USA). Statistical significance was defined as a two-sided p-value of < 0.05.

## Results

### Patient characteristics

**Figure 1:**
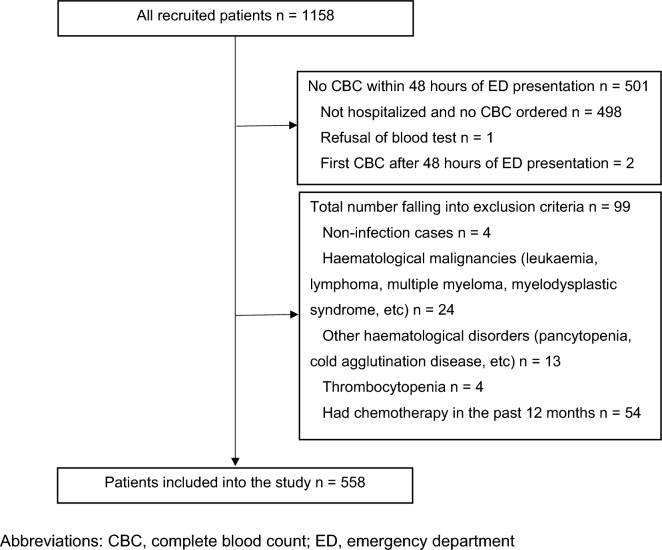
Patient flow diagram. CBC, complete blood count; ED, emergency department.

A total of 1158 adult patients met the initial screening criteria, but 501 were excluded because they did not have a CBC collected within 48 h of ED presentation, four were excluded because they were subsequently found to have a diagnosis other than an infection, 24 were excluded because of pre-existing hematological malignancies, 17 were excluded due to pre-existing hematological disorders (including four with pre-existing thrombocytopenia), and 54 were excluded because they had received chemotherapy in the past 12 months. In total, 558 patients were included and analyzed. The patient flow diagram is shown in Fig. 1.

Table 1 summarizes the patient demographic and clinical characteristics. The median age of the patients was 79.0 years (IQR 65.0–89.3), with males accounting for 49.1% and females accounting for 50.9%. The median CCI was 2 (IQR 0–3); of the 15 comorbidities included, dementia was the most common (n = 150, 26.9%), followed by diabetes mellitus with end organ damage (n = 149, 26.7%) and cerebrovascular accident or transient ischemic attack (n = 126, 22.6%). Concerning the infection, the respiratory system was the most frequently involved (n = 180, 32.3%), followed by the genitourinary system (n = 98, 17.6%) and the gastrointestinal system or intra-abdominal infections (n = 92, 16.5%). Regarding the pathogens, gram-negative bacteria were the most commonly found in bacterial cultures (n = 103, 18.5%), followed by mixed-organism infections (n = 99, 17.7%) and gram-positive bacteria (n = 43, 7.7%). Sixteen patients had COVID-19 (2.9%).

**Table 1:** Patient demographic, clinical and outcome characteristics.

Variables	All n = 558
Demographics	
Age—years, median (IQR)	79.0 (65.0–89.3)
Sex	
Male	274 (49.1)
Female	284 (50.9)
Old age home residency	212 (38.0)
Charlson Comorbidity Index—median (IQR)	2 (0–3)
Myocardial infarction	42 (7.5)
Congestive heart failure	49 (8.8)
Peripheral vascular disease	18 (3.2)
Cerebrovascular accident/transient ischaemic attack	126 (22.6)
Dementia	150 (26.9)
Chronic obstructive pulmonary disease	23 (4.1)
Connective tissue disease	9 (1.6)
Peptic ulcer disease	59 (10.6)
Liver disease	12 (2.2)
Drug controlled diabetes mellitus/with end organ damage	149 (26.7)
Hemiplegia	41 (7.3)
Moderate to severe chronic kidney disease	35 (6.3)
Solid tumour	91 (16.3)
Acquired immunodeficiency syndrome	1 (0.2)
Organ system involved	
Respiratory	180 (32.3)
Gastrointestinal/Intra-abdominal	92 (16.5)
Genitourinary	98 (17.6)
Soft tissue	42 (7.5)
COVID-19	16 (2.9)
Unknown	109 (19.5)
Pathogen	
Gram-positive bacteria	43 (7.7)
Gram-negative bacteria	103 (18.5)
Mixed organisms	99 (17.7)
SARS-CoV-2	16 (2.9)
Sepsis (SOFA ≥ 2)	144 (25.8)
ICU admission	7 (1.3)
Outcomes	
30-day mortality	60 (10.8)
2-day mortality	8 (1.4)
7-day mortality	17 (3.0)
90-day mortality	113 (20.3)

IQR, interquartile range; SOFA, sequential organ failure assessment score.

Based on the Sepsis-3 definition of sepsis, 144 (25.8%) of the patients had organ dysfunction, characterized by an increase in SOFA of ≥ 2 during the index acute hospital stay. Seven patients were admitted to the ICU. The 2- and 7-day mortality rates were 1.4% and 3.0%, respectively; 60 patients (10.8%) died by 30 days, and 113 patients (20.3%) died by 90 days.

### Comparing survivors vs. non-survivors at 30 days

We compared the patient demographics, clinical characteristics, and CBC parameters between the 498 survivors and 60 non-survivors by 30 days (Table 2). The non-survivors were significantly older (median age 89.0 vs. 77.0 years, p < 0.001) and more likely to be men (p = 0.039). A significantly higher proportion of non-survivors were old age home residents (p < 0.001) and had a higher CCI (p < 0.001). Non-survivors also had a significantly higher triage RR (p < 0.001), a lower SpO_2_ (p < 0.001), a lower MAP (p = 0.046), and a higher pulse rate (p = 0.045). Among all CBC parameters, the presenting hemoglobin level, hematocrit, RDW, total white cell, neutrophil, and lymphocyte counts differed significantly between survivors and non-survivors. Non-survivors had a significantly higher NLR (p = 0.001) and PLR (p = 0.007). Organ dysfunction was more common in non-survivors, whose worst SOFA score within 48 h of admission was significantly higher (median 2 vs. 0, p < 0.001). Table 3 shows the distribution of individual organ dysfunction among survivors and non-survivours.

**Table 2:** Comparison of patients who survived at 30 days and those who did not.

Variables	Survivors at 30 days n = 498	Non-survivors at 30 days n = 60	P value
Demographics			
Age—years, median (IQR)	77.0 (62.0–89.0)	89.0 (81.3–92.0)	< 0.001
Sex			
Male	237 (47.6)	37 (61.7)	0.039
Female	261 (52.4)	23 (38.3)	
Old age home residency	176 (35.3)	36 (60.0)	< 0.001
Charlson Comorbidity Index—median (IQR)	1 (0–3)	2 (1–5)	0.037
Triage clinical parameters—median (IQR)			
Temperature, ºC	37.8 (36.8–38.5)	37.6 (36.8–38.6)	0.650
Systolic blood pressure, mmHg	145 (126–164)	141 (117–154)	0.112
Diastolic blood pressure, mmHg	75 (64–87)	72 (62–81)	0.063
Mean arterial pressure, mmHg	99 (87–111)	95 (83–105)	0.046
Pulse rate, beats per minute	96 (82–107)	99 (90–114)	0.045
Respiratory rate, breaths per minute	16 (16–18)	18 (16–24)	< 0.001
Oxygen saturation, %	97 (96–98)	96 (94–98)	< 0.001
Complete blood count parameters—median (IQR)			
Total white blood cell count, × 10^3^/μL	10.4 (7.9–14.7)	13.3 (9.3–20.0)	0.002
Neutrophil, × 10^3^/μL	8.1 (5.7–12.7)	10.7 (6.8–18.8)	0.001
Lymphocyte, × 10^3^/μL	1.1 (0.7–1.6)	1.0 (0.6–1.2)	0.010
Monocyte, × 10^3^/μL	0.75 (0.54–1.05)	0.76 (0.43–1.14)	0.946
Basophil, × 10^3^/μL	0.03 (0.02–0.05)	0.04 (0.02–0.05)	0.161
Platelet count, × 10^3^/μL	232 (179–294)	260 (178–319)	0.246
Hemoglobin, g/dL	11.5 (10.2–13.2)	10.5 (9.0–12.2)	0.001
Hematocrit, %	0.34 (0.31–0.39)	0.32 (0.27–0.36)	0.001
Red Cell Distribution Width, %	13.2 (12.5–14.6)	13.9 (12.8–15.5)	0.018
Neutrophil–Lymphocyte Ratio	7.4 (4.1–13.9)	11.3 (6.9–25.6)	0.001
Monocyte-Lymphocyte Ratio	0.7 (0.4–1.1)	0.8 (0.5–1.1)	0.164
Platelet-Lymphocyte Ratio	208.2 (140.2–312.3)	275.9 (171.7–450.1)	0.007
Other information			
qSOFA score—median (IQR)	0 (0–0)	0 (0–1)	< 0.001
Sepsis (SOFA ≥ 2)	105 (21.1)	39 (65.0)	< 0.001
Worst SOFA score within 48 h of admission—median (IQR)	0 (0–1)	2 (1–4)	< 0.001
ICU admission	5 (1.0)	2 (3.3)	0.168

ICU, intensive care unit; IQR, interquartile range; qSOFA, quick Sequential Organ Failure Assessment; SOFA, Sequential Organ Failure Assessment.

**Table 3:** Individual organ dysfunction of patients who survived and who did not.

SOFA score components	Survivors at 30 days n = 498	Non-survivors at 30 days n = 60
Respiratory	0 (0–0)	1 (0–2)
PaO_2_/FiO_2_	339.3 (254.4–457.2)	159.0 (74.1–263.6)
SpO_2_/FiO_2_	457.1 (447.6–466.7)	366.7 (287.9–457.1)
Coagulation	0 (0–0)	0 (0–0)
Platelet count, × 10^3^/μL	229 (174–291)	259 (160–302)
Liver	0 (0–0)	0 (0–0)
Bilirubin, μmol/L	9 (6–13)	9 (6–18)
Cardiovascular	0 (0–0)	0 (0–0)
Central nervous system	0 (0–0)	0 (0–1)
Glasgow Coma Score	15 (15–15)	15 (10–15)
Renal	0 (0–0)	0 (0–1)
Creatinine, μmol/L	85 (63–121)	103 (67–171)

SOFA, sequential organ failure assessment.

Medians (interquartile range) are shown in the table unless otherwise stated.

### Discriminatory values of NLR, MLR, PLR, and their combination in predicting 30-day all-cause mortality

**Figure 2:**
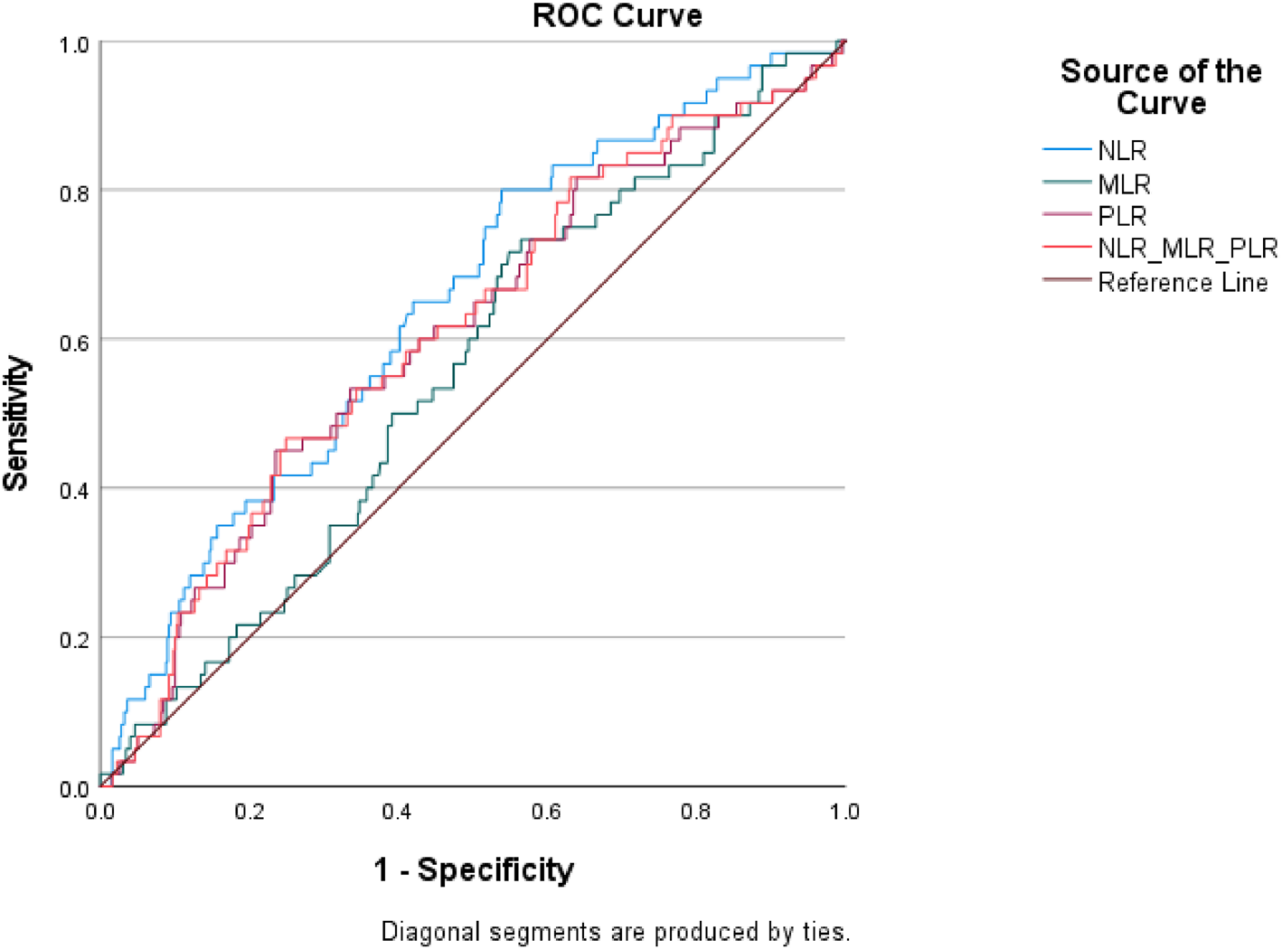
The discriminatory performance of NLR, MLR, PLR and their sum in sepsis prognostication. The AUROCs of NLR, MLR, PLR and the sum of NLR, MLR and PLR in predicting 30-day mortality in adult ED patients with infection were 0.644 (95% CI 0.573¬0.716), 0.555 (95% CI 0.482–0.628), 0.606 (95% CI 0.529–0.682), and 0.610 (95% CI 0.534–0.686), respectively.

In predicting 30-day all-cause mortality, the AUROCs of NLR, MLR, and PLR were 0.644 (95% confidence interval [CI] 0.573–0.716), 0.555 (95% CI 0.482–0.628), and 0.606 (95% CI 0.529–0.682), respectively. The AUROC of the sum of NLR, MLR, and PLR in predicting 30-day all-cause mortality was modest (0.610, 95% CI 0.534–0.686; Fig. 2).

### ANN in predicting 30-day all-cause mortality

**Figure 3:**
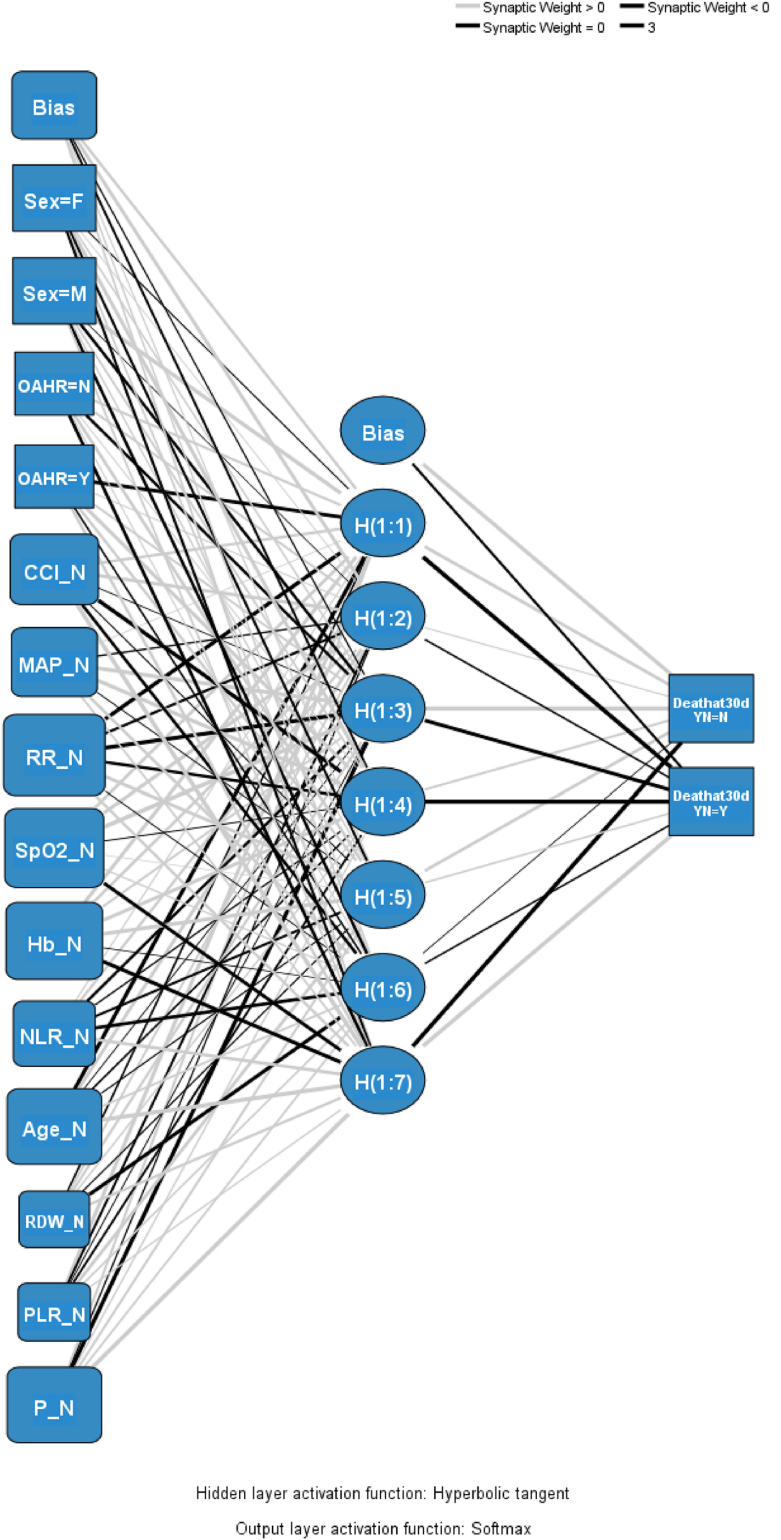
Architecture of the artificial neural network with twelve input variables, one hidden layer, and seven hidden nodes.

**Figure 4:**
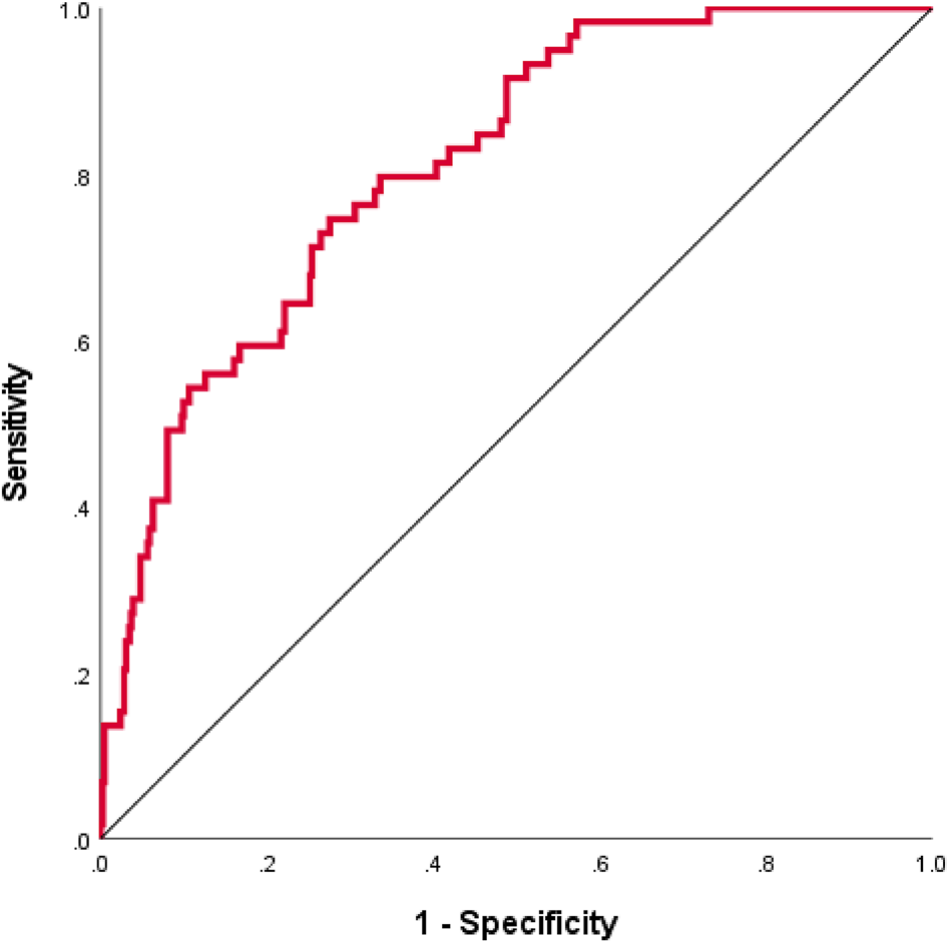
The discriminatory performance of the artificial neural network. The area under the receiver operating characteristic curve of the artificial neural network in predicting 30-day mortality was 0.811 in the testing dataset. CCI, Charlson Comorbidity Index; Hb, hemoglobin; MAP, mean arterial pressure; NLR, neutrophil-to-lymphocyte ratio; OAHR, old age home resident; P, pulse rate; PLR, platelet-to-lymphocyte ratio; RDW, red cell distribution width; RR, respiratory rate; SpO_2_, oxygen saturation.

**Figure 5:**
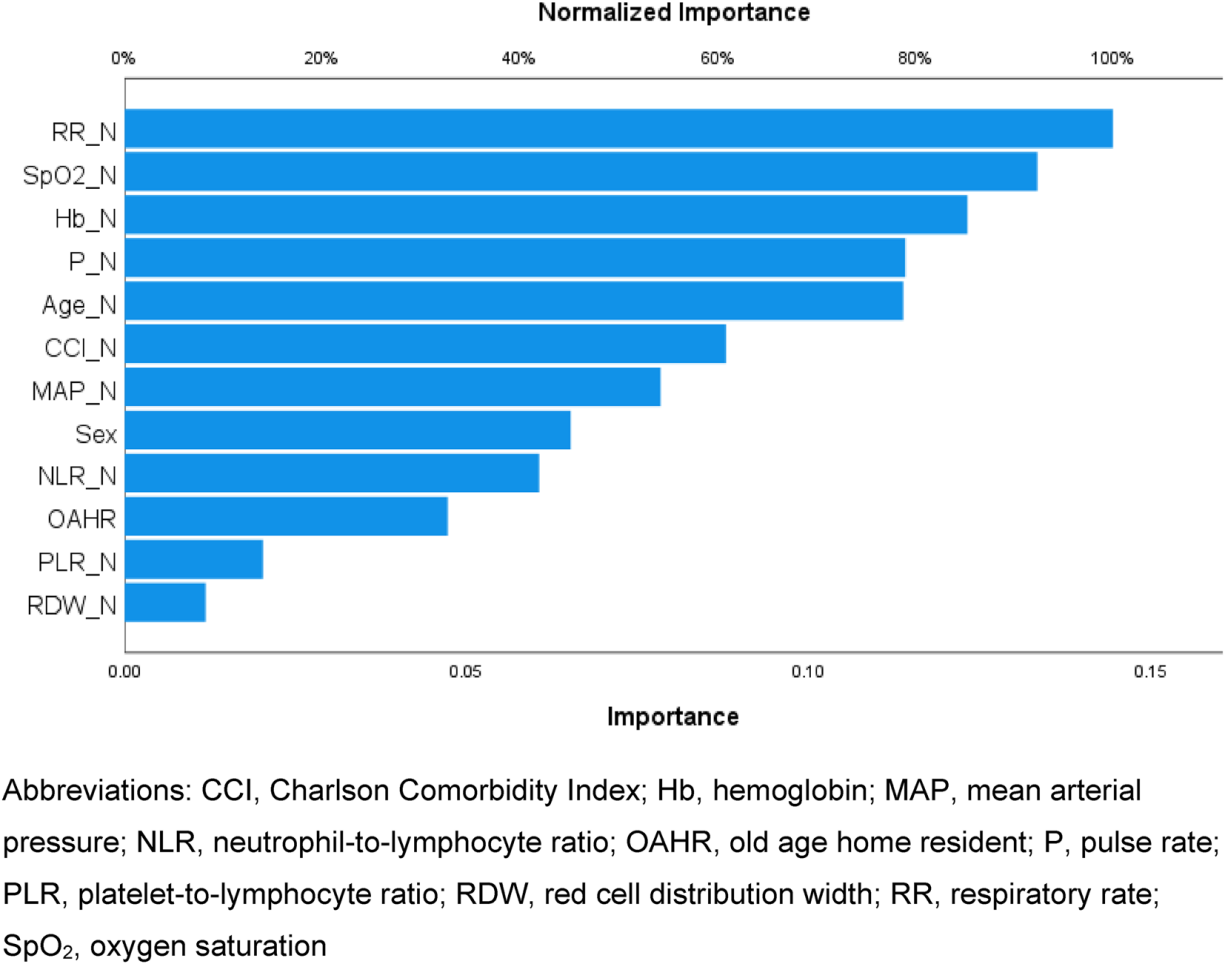
The relative normalized importance of individual input variables in the artificial neural network in predicting 30-day mortality of emergency department patients with infections.

Twelve variables with a significant association with 30-day all-cause mortality in univariate analyses, including patient age, sex, old age home resident status, CCI, triage MAP, pulse rate, RR, SpO_2_, hemoglobin, RDW, NLR and PLR, were inputted to the ANN model. The model has 1 hidden layer and seven nodes and its architecture is shown in Fig. 3. Table 4 shows the parameters of the model. The ANN model achieved an overall accuracy of 89.0% and 87.1% in predicting 30-day mortality in the training and testing datasets, respectively. The AUROC of the ANN model in the testing dataset was 0.811 (Fig. 4). The relative contributions of individual input variables in the ANN model are shown in Fig. 5. RR was the most important input variable, followed by SpO_2_, hemoglobin, pulse rate, and patient age.

**Table 4:** Parameters in the artificial neural network model.

Predictor		Predicted								
		Hidden Layer 1							Output layer	
		H(1:1)	H(1:2)	H(1:3)	H(1:4)	H(1:5)	H(1:6)	H(1:7)	[Death at 30d = N]	[Death at 30d = Y]
Input Layer	(Bias)	0.325	0.564	− 0.114	− 0.249	0.110	0.156	0.212		
	[Sex = F]	− 0.077	0.040	0.171	0.576	− 0.279	0.194	− 0.310		
	[Sex = M]	0.733	− 0.011	− 0.339	0.223	0.152	− 0.342	0.213		
	[OAHR = N]	0.274	0.236	− 0.332	0.061	0.352	0.076	− 0.412		
	[OAHR = Y]	− 0.616	0.073	0.233	0.611	0.314	− 0.172	0.330		
	CCI	0.326	0.444	− 0.040	− 0.819	− 0.070	− 0.344	0.888		
	MAP	0.015	− 0.156	0.727	0.678	− 0.061	0.339	0.196		
	RR	− 0.768	− 0.305	− 0.830	− 0.341	0.472	− 0.048	0.316		
	SpO_2_	0.361	0.595	0.912	− 0.063	0.002	0.124	− 0.570		
	Hb	0.588	0.335	0.235	0.529	0.706	− 0.015	− 0.699		
	NLR	0.086	0.307	− 0.327	− 0.122	− 0.260	− 0.496	0.382		
	Age	− 0.757	0.451	− 0.042	− 0.186	− 0.083	0.227	0.884		
	RDW	− 0.188	0.228	0.286	0.156	− 0.095	− 0.649	0.310		
	PLR	0.623	− 0.256	− 0.061	− 0.232	0.377	0.187	0.123		
	P	0.273	− 0.116	− 0.858	0.155	0.254	0.198	0.904		
Hidden Layer 1	(Bias)								0.718	− 0.291
	H(1:1)								0.401	− 1.035
	H(1:2)								0.082	− 0.172
	H(1:3)								1.235	− 0.664
	H(1:4)								0.243	− 0.872
	H(1:5)								0.343	0.180
	H(1:6)								− 0.028	− 0.163
	H(1:7)								− 0.965	0.870

CCI, Charlson Comorbidity Index; F, female; Hb, hemoglobin; M, male; MAP, mean arterial pressure; NLR, neutrophil-to-lymphocyte ratio; OAHR, old age home resident; P, pulse rate; PLR, platelet-to-lymphocyte ratio; RDW, red cell distribution width; RR, respiratory rate; SpO_2_, oxygen saturation.

## Discussion

Our study showed that 30-day survivors and non-survivors of infection differed significantly in terms of age, sex, old age home resident status, CCI, triage RR, SpO_2_, MAP, pulse rate, hemoglobin, RDW, NLR, and PLR on presentation. An ANN model incorporating these twelve input variables could achieve good discriminatory performance in predicting 30-day all-cause mortality in the testing dataset, compared with the modest discriminatory performance of NLR, MLR, PLR, and their sum.

The findings on the red cell parameters, including hemoglobin, hematocrit, and RDW, in predicting sepsis mortality are consistent with published studies. Jung et al. showed that initial hemoglobin levels were an independent factor of 90-day mortality, with odds ratios of 2.35, 1.97, and 1.88 for hemoglobin levels of < 7.0 g/dL, 7.0–7.9 g/dL, and 8.0–8.9 g/dL, respectively^6^. A retrospective study of 2,057 patients demonstrated that a low hematocrit level was an independent risk factor for 30-day mortality in septic patients. Luo et al. divided patients into low, regular, and high hematocrit levels, and the group with low hematocrit levels had a hazard ratio (HR) of 1.369 in univariate analysis and 1.589 in multivariate analysis^7^. A meta-analysis that involved 11 studies and 17,961 patients showed that an increased baseline RDW was associated with mortality (hazard ratio = 1.14)^8^. Since hemoglobin and hematocrit levels are highly correlated with each other, we only inputted the hemoglobin level to the ANN to avoid duplication.

As for the ratios between different differential WBC counts, the NLR appeared to have a better discriminatory performance in predicting 30-day mortality in our study compared with MLR and PLR. An elevated NLR may reflect physiologic stress that increases the number of neutrophil and decreases the number of lymphocytes. Sepsis also stimulates apoptosis of lymphocytes and 90–95% of septic patients have a NLR of > 3. When using the cutoff value of NLR > 10, it has a specificity of approximately 65% across several studies^20^. Djordjevic et al. found the AUROC of NLR as 0.681^12^, which is similar to our finding of 0.644 for NLR alone. In contrast, we found that the MLR had suboptimal discriminatory performance (AUROC 0.555), which is much lower than the AUROC of 0.655 reported by Li et al. in a retrospective study of 43,174 critically ill patients. However, this might be explained by differences in the study populations. Not all the patients in our study were critically ill^21^.

Regarding the PLR, we found that the ratio was significantly higher in non-survivors compared with survivors at 30 days. Interestingly, a meta-analysis involving 2,403 septic patients showed that PLR levels were significantly higher in non-survivors than survivors (random effect model: standardized mean difference [SMD] = 0.72, 95% CI; 0.35–1.10, p < 0.001). However, in the subgroup analysis of 1-month mortality, a significant difference was not demonstrated (SMD = 1.03, 95% CI = 0.15–1.92, p = 0.22)^11^. Similarly, we found that the AUROC of the PLR in predicting 30-day mortality was 0.606, indicating that the PLR alone has limited role in sepsis mortality prediction.

To improve the discriminatory performance of CBC parameters in predicting sepsis morality, attempts have been made to develop composite scores by combining different parameters. Djordjevic et al.^12^ showed that a composite bio score of MPV/PC, NLR, MLR, and PLR improved the AUROC to 0.718–0.874 in subgroups of patients with bacteremia. However, we could not replicate this method in our study because MPV is not reported in the routine CBC in our hospital. Another scoring system developed by Kim et al.^13^ that combined RDW, DNI, and MPV/PC had an AUROC of 0.67 in predicting 28-day mortality for septic patients, which was better than solely using the platelet count. However, DNI requires neutrophil data that are spaced 24 h apart, making it less relevant in clinical decision-making in the ED setting.

Instead of combining different CBC parameters, we found that combining the hemoglobulin level, RDW, NLR, and PLR on presentation with patient characteristics and triage vital sign variables could achieve a good discriminatory performance in predicting 30-day mortality using an ANN. This finding can be compared with other existing methods in sepsis prognostication. The performance of qSOFA was modest, with a pooled AUROC of 0.694 (95% CI 0.669–0.720) in predicting 30-day mortality across 29 studies of 108,122 patients. The pooled sensitivity over 60 studies in predicting in-hospital and 30-day mortality was 56.39%, and the pooled specificity was 74.58%^22^. Procalcitonin (PCT) and C-reactive protein (CRP) are commonly used biomarkers for sepsis. A meta-analysis of 23 studies involving 3,994 patients demonstrated that an elevated PCT level was associated with a higher mortality risk. The pooled relative risk was 2.60 (95% CI 2.05–3.30), and the AUROC was 0.77 (95% CI 0.73–0.80)^23^. Another meta-analysis involving 2,477 patients with community-acquired pneumonia showed that CRP had an AUROC of 0.62 (95% CI 0.58–0.67) in predicting in-hospital or 30-day mortality^24^. Our ANN model showed a comparable AUROC with PCT and represented an alternative method of sepsis prognostication without the need for additional biomarker testing. This finding has implications for sepsis care in resource-poor settings where access to procalcitonin or other sepsis biomarkers is limited.

The study has several limitations. Firstly, it was a single-center retrospective study with data collected during the winter months in Hong Kong. The findings might not be generalizable to other centers or ED patients who present in other seasons. Secondly, CBCs were not available for 501 patients, most of whom had mild infections not requiring hospital admission. The included patients were sicker, and a selection bias may have occurred. However, the need for early sepsis prognostication is higher among sicker patients with infections in the ED compared with those with mild infections. Thirdly, the time of blood-taking was not standardized, and the CBC data represented values at different times along the trajectory of sepsis progression. Lastly, we did not validate the derived ANN model in a separate cohort of patients. This warrants further large-scale out-of-the-sample validation before clinical application.

In summary, an ANN model that combined presenting hemoglobin, RDW, NLR, PLR, and clinical parameters showed better discriminatory performance than individual CBC parameters in predicting 30-day mortality in adult ED patients with an infection. Further validation studies in larger cohorts are necessary before clinical adoption.

## Data Availability

The dataset generated during and/or analysed during the current study are not publicly available due to local regulations on patient privacy but de-identified data are available from the corresponding author on reasonable request.
